# Supplementary material for: Willingness to help climate migrants: A survey experiment in the Korail slum of Dhaka, Bangladesh
Source: PLoS One. 2021 Apr 22;16(4):e0249315. doi: 10.1371/journal.pone.0249315 (PMC8062004; doi:10.1371/journal.pone.0249315)
Supplement: S1 Appendix — (DOCX) [file pone.0249315.s001.docx]

**S1 Appendix. Survey Questionnaire**

**Generic Frame:**

There is a very large number of poor people in urgent need of humanitarian assistance. For a variety of reasons, many people are forced to leave their homes and move to other areas in search for a better future or a safer place. However, they have very few resources and are often in dire poverty in temporary shelters, without even electricity. They are in urgent need of humanitarian assistance. While the government is providing many of these services, they are inadequate. Hence, several charities, such as the Bengal humanitarian organization, have stepped in to provide services like healthcare. Healthcare relief could include assistance with medicines, doctor’s fees, or emergency medical services.

**Persecuted Migrants Frame:**

There is a very large number of poor people in urgent need of humanitarian assistance. For example, religious violence is causing a large displacement of people, forces many to leave their homes and move to other areas in search for a better future or a safer place. However, they have very few resources and are often in dire poverty in temporary shelters without even electricity. While the government is providing many of these services, they are inadequate. Hence, several charities, such as Bengal humanitarian organization, have stepped in to provide services like healthcare. Healthcare relief could include assistance with medicines, doctor’s fees, or emergency medical services.

**Climate Change Migrants Frame:**

There is a very large number of poor people in urgent need of humanitarian assistance. For example, climate change is causing problems such as heavy flooding, which forces many to leave their homes and move to other areas for a better future or a safer place. However, they have very few resources and are often in dire poverty in temporary shelters, without even electricity. While the government is providing many of these services, they are inadequate. Hence, several charities, such as the Bengal humanitarian organization, have stepped in to provide services like healthcare. Healthcare relief could include assistance with medicines, doctor’s fees, or emergency medical services.

1. For reference, a typical family spent about 1,500 takas on medicine last month. Imagine that Bengal humanitarian organization contacted you to request a donation for the above mentioned refugee group. Would you be willing to donate100 takas?

1= No

2= probably not

3= maybe

4= probably yes

5= yes

*I will ask you some questions just to make sure that I correctly read aloud the survey to you clearly:*

1. What type of help does the Charity provide?
   1. Healthcare
   2. Job training
   3. Food assistance
   4. All of the above
2. The Charity serves elderly people only.

a. Yes

- 1. No

1. The charity has provided shelters with electricity only and nothing else.
   1. Yes
   2. No

*Now I would to ask for your opinion on some relevant issues:*

1. The most important issue facing Bangladesh is:
2. Unemployment
3. Climate change
4. Corruption
5. Political strife
6. Do you think crime in Bangladesh has increased in the last 10 years?
7. Yes
8. No

1. If answer to Q3 is yes; the reason crime has increased is:
2. Refugees
3. Corruption
4. Declining in morality

*I will now ask some questions about yourself:*

1. What is your gender?
2. Female
3. Male
4. Non-binary / Third gender
5. Prefer to self-describe
6. In what year were you born?

[ENTER YEAR]

1. What is your household monthly income?
   1. Above 12,000 BDT
   2. Between 9,000-12,000 BDT
   3. Between 5,500-8,999 BDT
   4. Between 1,500-5,499 BDT
   5. Less than 1,500 BDT
2. What is your marital status?
3. Single, never married
4. Married
5. Separated
6. Divorced
7. Widowed
8. How many children do you have?

[ENTER NUMBER]

1. What is your current occupation?
2. Work for pay (salary, wage, self-employed)
3. Work without pay (apprentice, family business)
4. Do not work but am looking for a job
5. Student
6. Retired
7. Homemaker
8. Unable to work
9. Other, please specify:
10. What is your religion?
11. Muslim
12. Hindu
13. Christian
14. Buddhist
15. Other, please specify:
16. Yesterday, did you:

|  | Yes | No |
| --- | --- | --- |
| a. Read a daily newspaper? |  |  |
| - 1. Watch the news or a news program on television? |  |  |
| - 1. Listen to any news on radio or FM radio? |  |  |
| - 1. Read/watch news on your phone (e.g., Facebook, online portal etc.) or computer? |  |  |

1. How many years have you lived in Dhaka?

[ENTER YEARS]

1. Have you or your family experienced floods in the last year?
   1. Yes
   2. No
