# Supplementary figures and images for: Willingness to help climate migrants: A survey experiment in the Korail slum of Dhaka, Bangladesh
Source: PLoS One. 2021 Apr 22;16(4):e0249315. doi: 10.1371/journal.pone.0249315 (PMC8062004; doi:10.1371/journal.pone.0249315)

**S2 Appendix. OLS Regression**


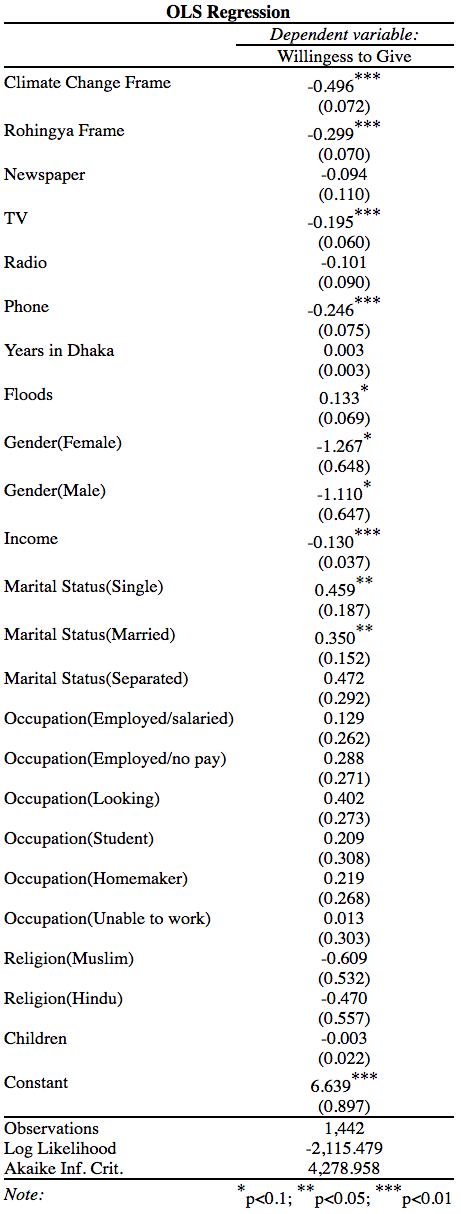

Supplement: S2 Appendix — (DOCX) [file pone.0249315.s002.docx]

**S3 Appendix. Balance Table**


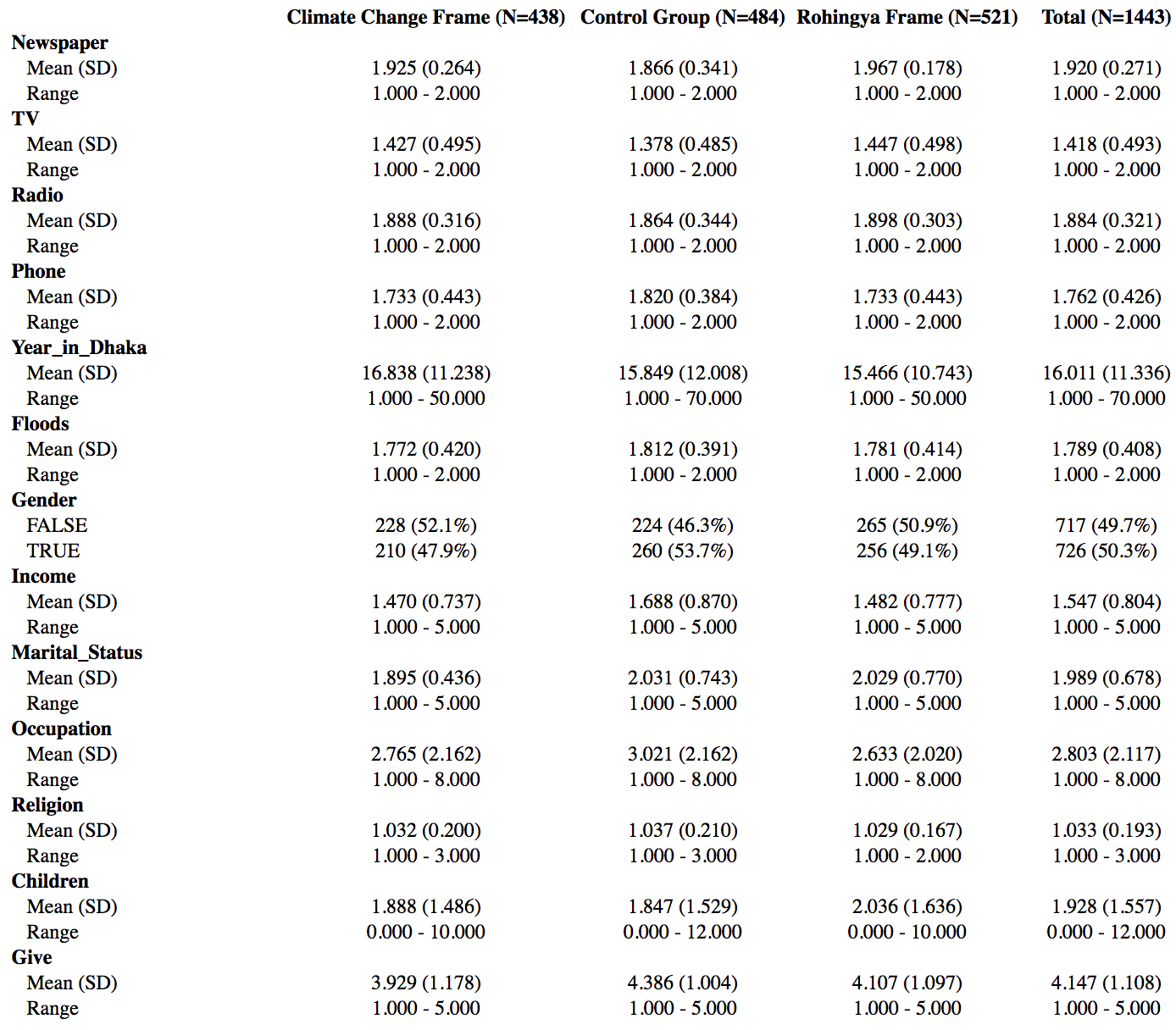

Supplement: S3 Appendix — (DOCX) [file pone.0249315.s003.docx]

**S4 Appendix. Ordered Probit Results**

**
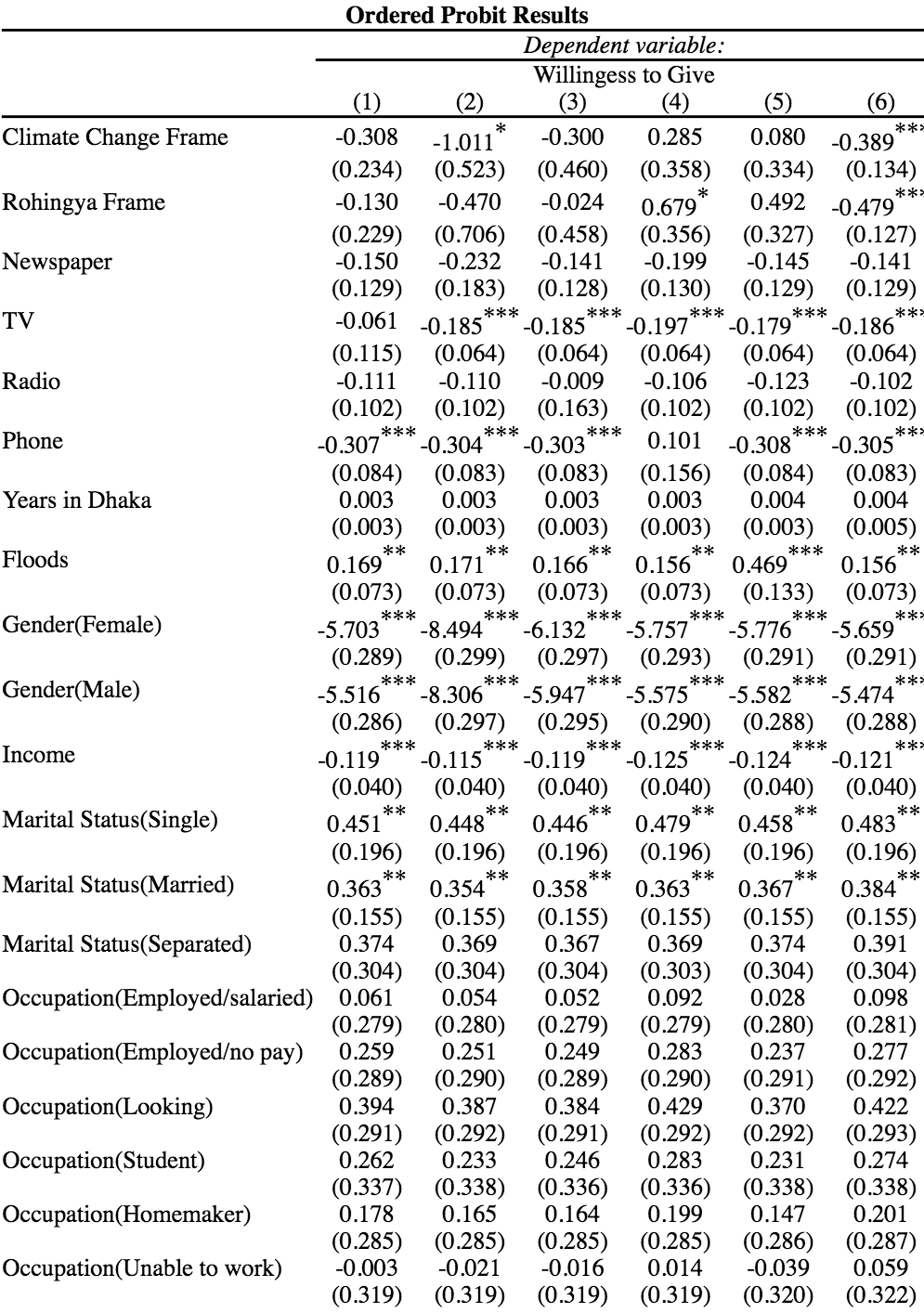
**

**
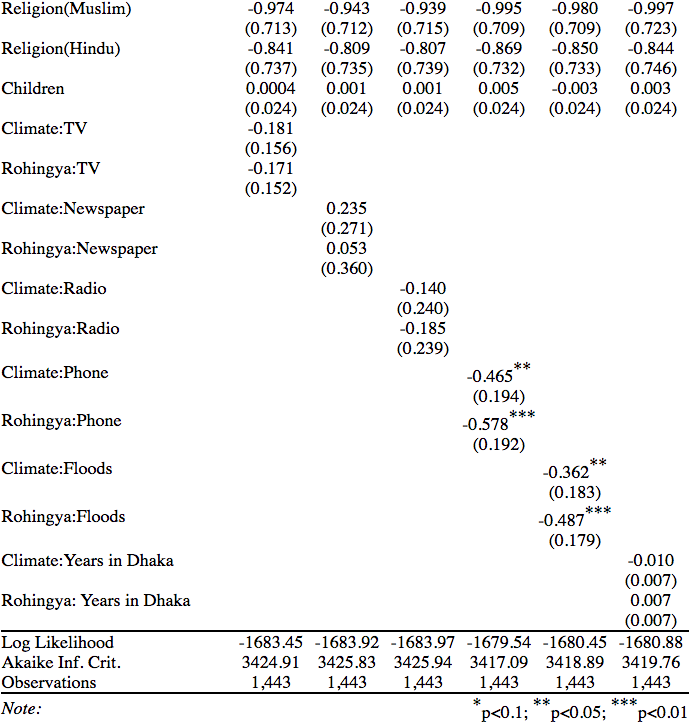
**

Supplement: S4 Appendix — (DOCX) [file pone.0249315.s004.docx]

**S5 Appendix. Ordered Probit Results – Full Sample**


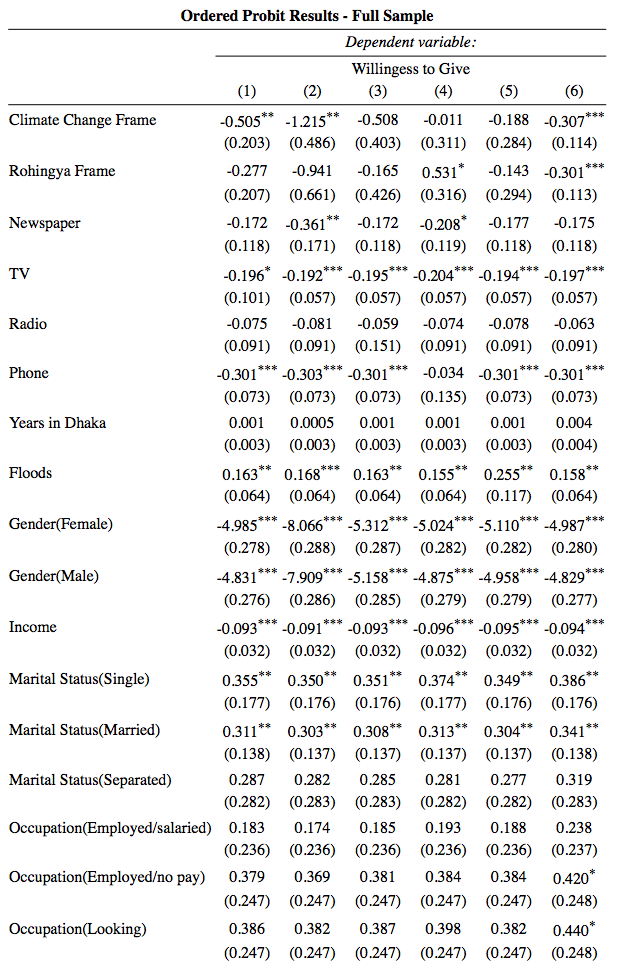


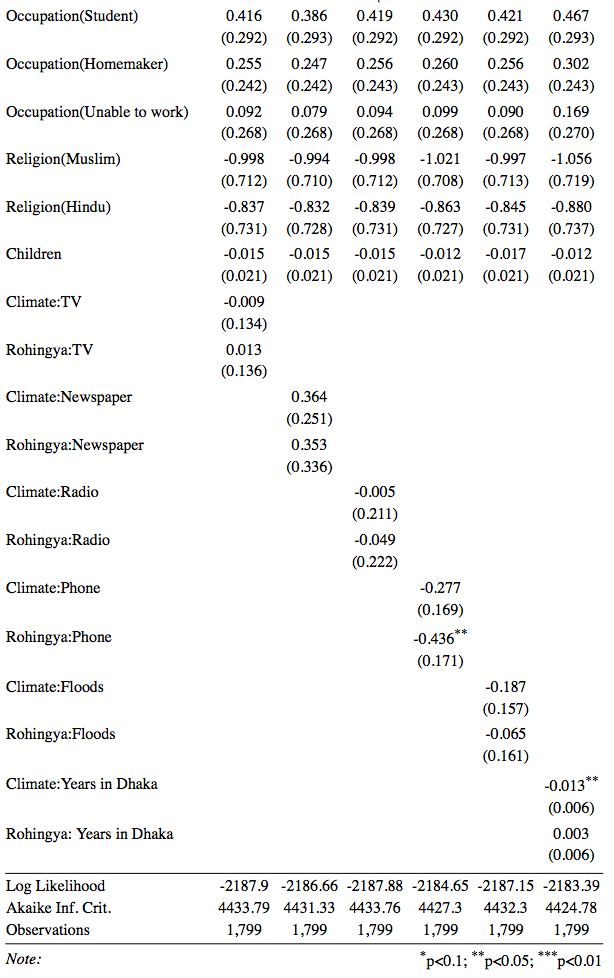

Supplement: S5 Appendix — (DOCX) [file pone.0249315.s005.docx]
