# Supplementary material for: Willingness to help climate migrants: A survey experiment in the Korail slum of Dhaka, Bangladesh
Source: PLoS One. 2021 Apr 22;16(4):e0249315. doi: 10.1371/journal.pone.0249315 (PMC8062004; doi:10.1371/journal.pone.0249315)
Supplement: S6 Appendix — (DOCX) [file pone.0249315.s006.docx]

**S6 Appendix. Demographic Profile of Survey Participants**

|  | **Percentage of Survey Participants** |
| --- | --- |
| **Religion** |  |
| Muslim | 97.3% |
| Hindu | 2.5% |
| Christian | 0.2% |
|  |  |
| **Gender** |  |
| Male | 50% |
| Female | 50% |
|  |  |
| **Employment** |  |
| Employed | 46.7 % |
| Homemakers | 21.5 % |
| Unemployed but Looking | 12.2 % |
|  |  |
| **Marital Status** |  |
| Single, never married | 13.4 % |
| Married | 81.3 % |
| Separated | 1.3 % |
| Divorced | 1 % |
| Widowed | 3 % |
|  |  |
| **Monthly Household Income** |  |
| Above 12,000 BDT | 60.2 % |
| 9,000-12,000 BDT | 28.8 % |
| 5,500-8,999 BDT | 7.9 % |
| 1,500-5,499 | 2.2 % |
| Less than 1,5000 BDT | 0.9 % |
