# Supplementary material for: Willingness to help climate migrants: A survey experiment in the Korail slum of Dhaka, Bangladesh
Source: PLoS One. 2021 Apr 22;16(4):e0249315. doi: 10.1371/journal.pone.0249315 (PMC8062004; doi:10.1371/journal.pone.0249315)
Supplement: Attachment — Submitted filename: PLOS ONE Response to reviewers march 7 2021.docx [file pone.0249315.s008.docx]

March 7, 2021

Dr. Bernhard Reinsberg, Ph.D

Academic Editor

*PLOS ONE*

Dear Dr. Reinsberg,

Thank you for the opportunity to re-revise and resubmit our manuscript, “Willingness to help climate migrants: A survey experiment in the Korail Slum of Dhaka, Bangladesh” (PONE-D-20-35549R1). We thank the reviewers again for their feedback. We are enclosing a rebuttal memo detailing their suggestions and outlining specific ways we address them in the revised manuscript.

In your letter, you advised us to “back up your assumptions about how individuals learn about policy positions of their government regarding climate migrants. Furthermore, you should check your survey for cues from respondents in the control group about what they think when thinking about migrants in general.”

In this survey, we did not ask directly about how respondents learn about policy issues in general or about government’s positions on climate migrants. This is for two reasons. First, people typically get much of their policy information from different types of mass and social media. Of course, we do not know if this information is authoritative and if they comprehend this information. Indeed, we are not making any claim on how well informed or poorly informed individuals are about climate change, migration, or any national policies. We recognize that as boundedly rational actors, individuals develop opinions about issues based on incomplete information. Yet, no matter how incomplete or poorly informed individuals are, public opinion matters. And this is what we examine in the context for public support for a charity that provides health services to climate migrants and Rohingyas (in relation to generic migrants).

Because mass and social media plays a crucial role in shaping public opinion, we have asked questions about respondent’s exposure to different types of media (Q15 of the survey):

a.	Read a daily newspaper?

b.	Watch the news or a news program on television?

c.	Listen to any news on radio or FM radio?

d.	Read/watch news on your phone (e.g., Facebook, online portal etc.) or computer?

We have analyzed if exposure to different media might influence respondent’s support for climate migrants or Rohingyas in relation to generic migrants. Anticipating some of concerns of the reviewer, we have not subsumed all types of media in category: we have examined them separately. The reason is that some respondents might rely on newspapers, and others on Facebook, to access policy information. Also, it is possible that some media might cover climate issues more extensively or effectively than others. For example, television might provide more content about challenges faced by climate migrants in relation to, say radio. Or, the television footage might create more empathy for climate migrants. If so, those with higher exposure to television might reveal higher support for the charity supporting climate migrants.

Also, please note that our model controls for factors such as prior experience with floods that might make them more prone to access or pay attention to specific type of climate information in any media. Finally, because this is a survey experiment in which respondents are randomly assigned to different frames, unobserved heterogeneity in respondents’ characteristics should not influence support for any frame.

When we rerun the analysis for each media type separately, our results do not change (i.e. support for climate migrants or Rohingyas in relation to the generic frame). This gives us additional confidence that the medium through which respondents might receive information is not changing the support for climate migrants in relation to generic migrant. We have included this discussion in the revised paper.

Climate migrants:

We have identified a recent paper which investigates whether respondents might perceive climate migrants differently from other migrants. In the context of Germany, Helbling (2020) reports that German survey respondents are more supportive of climate change migrants, in relation to economic migrants. We have referenced this paper in the revised manuscript.

We are enclosing the revised paper and the rebuttal memo. Please let us know if you need anything else from us.

Sincerely,

Rachel Castellano, University of Washington, Seattle

Nives Dolšak, University of Washington, Seattle

Aseem Prakash, University of Washington, Seattle

**Rebuttal Memo**

Willingness to help climate migrants:

A survey experiment in the Korail Slum of Dhaka, Bangladesh

PONE-D-20-35549R1

**Reviewer #2**

1. *This is a strong revision and I would be delighted to see this research published, pending a few minor revisions listed below. I paid particular attention to the author(s)’ responses to the prior referee reports, and to their alterations of the manuscript to reflect their adjustments to critiques. In particular, the theoretical framework in the revised manuscript is considerably strengthened, the link between theory and empirics is more focused, and the empirical evidence remains robust to the different sensitivity checks that the author(s) have now introduced. Overall, I am impressed by the constructive revisions undertaken by the author(s) and see the author(s) as admirably responsive to the prior concerns raised in review.*

Response:

Thank you.

Minor revisions:

1. *In l.106 “especially if it involves citizens to incur private costs” would read better as “especially if it involves citizens incurring private costs.”*

Response:

Done.

2. *In response to R1’s excellent comments, the author(s) now address the issue of religious similarities and differences between the migrants and respondents quite extensively in the manuscript, noting that the Rohingya share the same Islamic faith as the majority of Bangladeshis (e.g., p.10), that respondents would likely not have associated the migrants with religious minorities like Hindus (p.12), and that respondents could have been expected to be “sympathetic to their co-religionists” (p.14, l.304).* *In neighboring India, which is frequently referenced in the manuscript, existing public opinion work shows that city residents belonging to the majority religious group (in the Indian case, Hindus) do not discriminate again rural-to-urban migrants based on religious profiles. Instead, residents from the dominant religious group appear to care mainly about the economic impact of migration. This is in fact consistent with what the author(s) find in the Bangladesh context: slum residents do not appear to heed religious concerns in responding to migration. The manuscript would be strengthened by citing and referencing these points as it would link the manuscript to broader debates in comparative politics.*

*Citation:* *Gaikwad, N. and Nellis, G. (2017), “The Majority‐Minority Divide in Attitudes toward Internal Migration: Evidence from Mumbai.” American Journal of Political Science, 61: 456-472*.

Response:

Thanks for suggesting this article which we have referenced in the revised paper.

3. *In l.343, the sentence is missing a closed parenthesis mark.*

Response:

Corrected.

*Overall, I continue to believe that this manuscript tackles a very important subject, and I expect it to foreshadow new work on climate change and migration.*

Response:

Thank you*.*

**Reviewer #3**

1. *This paper examines individuals’ willingness to provide assistance to climate migrants as compared to other types of migrants by means of a survey-embedded experiment conducted in a slum in Dhaka. The paper is well-written and addresses an important and very timely question. I believe that this study can make a contribution to the literature after addressing the issues below*.

Response:

Thank you.

Theory

2. *I think the authors need to provide a more specific discussion of the mechanism(s) underlying their main hypothesis, especially “H1: Survey respondents will be more willing to support climate migrants in relation to generic migrants.” The arguments that the authors provide (line 222: “extensive focus on climate change in media and the strong advocacy by the Bangladesh government in global forums”) are based on the assumption that citizens of Korail slum take their cues from the media and policymakers and will formulate their willingness to support (climate) migrants accordingly. However, this not only requires that slum residents have access to these specific information, but are also well-informed about what their national politicians advocate in global policy arenas.*

Response:

In this survey, we did not ask directly about how respondents learn about policy issues in general or about government’s positions on climate migrants. This is for two reasons. First, people typically get much of their policy information from different types of mass and social media. Of course, we do not know if this information is authoritative and if they comprehend this information. Indeed, we are not making any claim on how well informed or poorly informed individuals are about climate change, migration, or any national policies. We recognize that as boundedly rational actors, individuals develop opinions about issues based on incomplete information. Yet, no matter how incomplete or poorly informed individuals are, public opinion matters. And this is what we examine in the context for public support for a charity that provides health services to climate migrants and Rohingyas (in relation to generic migrants).

Because mass and social media plays a crucial role in shaping public opinion, we have asked questions about respondent’s exposure to different types of media (Q15 of the survey):

a.	Read a daily newspaper?

b.	Watch the news or a news program on television?

c.	Listen to any news on radio or FM radio?

d.	Read/watch news on your phone (e.g., Facebook, online portal etc.) or computer?

We have analyzed if exposure to different media might influence respondent’s support for climate migrants or Rohingyas *in relation to* generic migrants. Anticipating some of concerns of the reviewer, we have not subsumed all types of media in category: we have examined them separately. The reason is that some respondents might rely on newspapers, and others on Facebook, to access policy information. Also, it is possible that some media might cover climate issues more extensively or effectively than others. For example, television might provide more content about challenges faced by climate migrants in relation to, say radio. Or, the television footage might create more empathy for climate migrants. If so, those with higher exposure to television might reveal higher support for the charity supporting climate migrants.

Also, please note that our model controls for factors such as prior experience with floods that might make respondents more (or less) prone to access or pay attention to specific type of climate information in any media. Finally, because this is a survey experiment in which respondents are randomly assigned to different frames, unobserved heterogeneity in respondents’ characteristics should not influence support for any frame.

When we rerun the analysis for each media type separately, our results do not change (i.e. support for climate migrants or Rohingyas in relation to the generic frame). This gives us additional confidence that the medium through which respondents might receive information is not changing the support for climate migrants in relation to generic migrant. We have included this discussion in the revised paper.

3.	*I think the authors could expend more efforts to provide more micro-level arguments as to why they think climate migrants might be perceived in more favorable lights than other (generic) migrant types*.

Response:

Fair point. The subject of public support for climate migration (in relation to other types of migration) is relatively new in the climate policy literature (although as we point out in the paper, there is extensive literature on levels of climate migration in parts of the world, and whether climate migrants should be recognized as refugees). We have identified a recent paper which investigates whether respondents might perceive climate migrants differently from other migrants. In the context of Germany, Hebling (2020) reports that German survey respondents are more supportive of climate change migrants, in relation to economic migrants. We have referenced this paper in the revised manuscript.

Helbling, M. (2020). Attitudes towards climate change migrants. *Climatic Change*, 1-14.

3. *My main issue with this manuscript is the design of the generic frame. The problem is that the lack of a specific driver of migration in this frame introduces* *the possibility for survey respondents to think about all sorts of migrant type, including climate migrants as well as both domestic and international migrants. I think this heterogeneity, but more importantly, the impossibility to identify what type of migrant the respondent is thinking about when answering whether they’d like to help that group of migrants, makes it very hard to interpret the outcome variable itself. What is the level of support we are benchmarking support for climate migrants against? We know from the literature that different types of migrants are seen in very different ways (as this manuscript also argues). Therefore, it is important to know how individuals perceive climate migrants compared to other (specific) types of migrants. I wonder whether the authors asked some open-ended questions about what types of migrants (or general thoughts) the respondents had in mind after reading the frames. This may give us some hint about the type of migrant (and potential biases/associations) people had in mind when reading the respective treatment text. At the least, this shortcoming should be addressed in greater detail in the manuscript.*

Response:

Migrants have different characteristics. The vast literature on migration studies has examined public support when specific characteristics of the emigrant such as religion, gender, skill level, etc. are highlighted. We contribute to this literature by focusing attention to a specific characteristic that the literature has overlooked: climate change as a migration driver. Thus, the generic frame does not highlight any migration driver unlike the two treatment frames. Consequently, this research design allows us to assess the change in public support when one specific migrant characteristic (migration driver: climate change or religious persecution) is highlighted in the two treatment frames while all other information remains the same as the generic frame.

This is also why we do not have any open-ended questions to investigate what types of migrants the respondents had in mind after reading the generic frame because we are not examining how respondents perceive generic migrants. Rather, we want to see how support for migrants might shift (in relation to the generic migrant) when one specific migration driver is highlighted. We have included a discussion on this subject in the manuscript.

4. *I have two concerns with the way that the outcome variable was formulated. First, the reference “a typical family spent about 1,500 takas on medicine last month” may have exacerbated social desirability bias, since it conveys the information that compared to what a typical family spends on healthcare, 100 takas are not much. Second, the way that this question is formulated, I wonder whether it may also have tapped into people’s support for NGOs more generally rather than just capturing people’s support for the specific migrant group described in the treatment text*.

Response:

We have acknowledged the issue of social desirability bias in the previous revision. If there is a bias, because respondents are randomly assigned to different groups, it cannot explain the difference in support between the generic and treatment frames.

Similarly, we do not have data to speculate whether respondents were responding to support for NGOs. All respondents were given the same information cues with only difference between the generic and treatment frames: drivers of migration. Again, because respondents were randomly assigned to different frames, how respondents interpreted the information cue is not important for this study.

5. *As an additional comment, I am curious why the authors did not use a continuous variable by asking respondents how much of the 100 takas they’d be willing to donate*.

Response:

We discussed this possibility of using some sort of a slider scale to ask respondents for their support for 100-taka donation on say 1-5 scale. Because this was an in-person survey (as opposed to an online one), the survey firm thought that the logistical issues will be difficult if we were to ask respondents to use the slider on the smartphone and might even be distracting. Hence, we decided to work with a 5-point Likert scale – which is consistent with most survey experiments in the climate policy field. In the Appendix, we also present our results using the OLS estimator, where the dependent variable is treated as continuous. We have noted this in the revised paper.

6. *The authors discuss how much the average Bangladeshi household spends on healthcare. Is this the same amount that the average slum resident in Korail spends on healthcare? In general, I would like to hear more about in what way the survey site (the Korail slum) differs from other slums, or the country*.

Response:

We do not have data on healthcare spending in Korail slum in relation to other slums. Also, the issue of level of healthcare expenditure is not critical to our research design. Across all frames, generic frame and the treatment frame, we had the same ask for donation, 100 takas. In the revised manuscript, we have noted that this research design pertains to only one “price point,” namely 100 takas. To further validate our study, future work could look at different price points. Also, our paper already acknowledges that this study should be replicated in other locations/slums to validate its findings.
